# The impact of allergen exposure and specific immunotherapy on circulating blood cells in allergic rhinitis

**DOI:** 10.1186/s40413-018-0197-0

**Published:** 2018-08-15

**Authors:** Galateja Jordakieva, Erika Jensen-Jarolim

**Affiliations:** 1 Department of Physical Medicine, Rehabilitation and Occupational Medicine, Medical University of Vienna, Spitalgasse 23, Vienna, 1090 Austria; 2 Department of Pathophysiology and Allergy Research, Institute of Pathophysiology and Allergy Research, Center of Pathophysiology, Infectiology and Immunology, Medical University Vienna, Spitalgasse 23, 1090 Vienna, Austria; 3 The interuniversity Messerli Research Institute, University of Veterinary Medicine Vienna, Medical University Vienna, University of Vienna, Veterinaerplatz 1, Vienna, 1210 Austria; 4 AllergyCare, Allergy Diagnosis and Study Center Vienna, Vienna, Austria

**Keywords:** Allergen challenge, Allergic rhinitis, Allergen-specific immunotherapy, Circulating blood cells, Differential blood count

## Abstract

Allergic rhinitis (AR) is an IgE-mediated inflammatory disease of the nasal mucosa with well described local immune responses during allergen exposure. The frequent association of AR with general extra-nasal symptoms and other allergic conditions, such as conjunctivitis and asthma, however, support a more systemic disease impact. In addition to acute elevation of soluble inflammatory mediators in periphery blood, a growing number of studies have reported changes in circulating blood cells after specific nasal allergen challenge or environmental allergen exposure. These findings imply an involvement of specific blood leukocyte subsets, thrombocytes and recently, erythrocytes. This review summarizes the circulating blood cell dynamics associated with allergen exposure in AR subjects reported so far. Additionally, the impact of therapy, particularly allergen-specific immunotherapy (AIT), the only currently available causal treatment reducing AR-related symptoms, is further considered in this context.

## Background

Allergic rhinitis (AR) refers to nasal mucosa inflammation upon repeated aeroallergen exposure in susceptible subjects [1]. The underlying **early** inflammatory response is characterized by IgE-mediated release of preformed histamine from activated mast cells, leading to up-regulation of pro-inflammatory cytokines and adhesion molecules. The **late** phase reaction is based on the release of newly generated mediators, such as leukotrienes (LT), and a wave of effector cells recruited from lymphatic tissue and circulating blood [2–4]. Indeed, circulating leukocytes and their subsets markedly differ in blood of symptomatic AR patients compared to healthy controls [5] and even an activation of platelets has been reported [6, 7]. Recently, we further described a rapid decrease in circulating erythrocytes after allergen exposure in AR [8, 9]. Thus, local nasal inflammation upon allergen exposure induces systemic inflammatory responses [10] and, in the context of effector cell recruitment, activation of bone marrow. This raises the question if changes in circulating blood cell numbers are indicative of the allergic nasal inflammatory response. The aim of this review was to summarize previously described effects of allergen exposure on complete and differential blood counts in adult patients suffering from AR and the observed impact of allergen-immunotherapy (AIT).

## Blood cells in AR

### T cells

**T helper cells** are the main players in the allergic airway inflammatory response. CD4+ T cells are characterized by specific surface molecules expression and cytokine secretion profiles [11]. **Th1 cells** result from an IL-12 and IFN-γ driven polarization of naïve CD4+ T cells (Th0) towards a Th1 phenotype. Lower Th1 numbers and thus, lower Th1-derived IFN-γ levels were found in nasal lavage fluids of AR patients compared to controls [12]. A higher IL-4 and lower IFN-γ production in cultured blood Th cells from pollen-sensitized seasonal AR patients is found compared to controls implying a Th2 skewing in allergic disease [13]. **Th2 cell** polarization in Th0 is mainly promoted by IL-4, but may also be induced by epithelium-damage associated release of TSLP, IL-25 and IL-33. Main effector cytokines of Th2 cells are IL-4, IL-5, and IL-13, supporting B cell-derived IgE production, eosinophil activation and mast cells-derived histamine and LT release. Th2 cytokines further suppress Th1 cell differentiation. Ratios of Th1/Th2 and T_reg_/Th2 are reduced in the nasal mucosa of AR patients during allergen season [14]. A relative predominance of Th2 cytokine profiles, except for IL-4 levels, was found in nasal fluid of olive-sensitized AR patients compared to healthy controls [12]. In circulating blood, Th2 cells are increased in AR patients while AIT has been shown to lower Th2 cell numbers in grass-pollen sensitized AR patients [15]. The **Th9 cell** subsets produce IL-9, IL-10 and IL-21 and are associated with eosinophil survival during allergic airway inflammation. In a recent murine model, a greater Th9 cell percentage, higher Th9 cell transcription factor mRNA and higher IL-9 levels were found in nasal mucosa of AR mice compared to controls. Further, after intranasal application of anti-IL-9 antibodies less eosinophil nasal mucosa infiltration was described [16]. Higher Th9 numbers were found in Chinese AR patients compared to controls. Further, IL-9 levels correlated with circulating eosinophil numbers and clinical symptom scores [17]. Another study did not observe different Th9 levels in HDM sensitized AR and controls [18]. AIT resulted in a lower frequency of Th2, Th9 and Th17 and higher values of Th1 and T_reg_ cells and their respective cytokines [19]. **Th17 cells** are generated upon TGFβ, IL-6 and IL-21 co-stimulation and produce IL-17A, IL-17F, IL-22 and IL-26, but also IL-6 and IL-21 as positive feed-back loop cytokines. IL-17 directly promotes neutrophil recruitment and indirectly supports neutrophil generation via GM-CSF production. In a murine AR model, antibody mediated neutralization of IL-17 resulted in less eosinophil and neutrophil infiltration and lower Th2 and Th17 cytokine levels in nasal mucosa [16]. Elevated Th17 percentages and serum IL-17A levels have been reported in AR patients compared to controls [18, 20, 21] and a reduction of circulating Th17 cells after successful AIT has been reported [19]. A more recent study did not observe changes in Th17 numbers and function in AR patients during pollen season [22]. **Tfh cells** and their main effector cytokine IL-21 induce generation of B memory cells and isotype class switching in plasma cells. Tfh cells produced IL-4 and induced effector Th2 cell responses after allergen challenge in HDM allergic subjects; further Th2 responses were shown to be impaired in Tfh cell absence [23]. IL-4-committed Tfh were described to be Th2 cell precursors in HDM associated allergic airway disease [24], indicating their role in supplying new effector cells after allergen encounter as memory Th cell reservoirs in secondary lymphoid organs [25]. However, while one study found reduced circulating Tfh in human AR [26], they did not differ in another study compared to healthy controls [27]. **Th22 cells** and their main effector cytokine IL-22, were higher in peripheral blood of HDM sensitized AR patients compared to controls correlating with clinical symptom scores [18]. **yδT cells,** secreting IFN-γ, IL-17A, IL-17F and IL-22, are usually enriched at epithelial surfaces and manifest both innate and adaptive immunity characteristics; they release IL-4 and IL-13 and induce B cell IgE synthesis. [28] In AR, yδT cells support IL-5, IL-13 and IFN-γ release in the Th2 inflammatory response [29]. Nasal yδT cells (Vg1/Vd1-Jd1 TCR+) seem to differ from those in peripheral blood (Vg2/Vd2 TCR+) [30], implying that nasal mucosa yδT cells are organ specific and proliferate locally in an oligoclonal fashion upon allergen exposure [28]. Still, higher yδT cell percentages were described in circulating blood of AR patients, correlating with Th17 numbers and levels of IL-17 [20]. A Chinese study found increased circulating yδT numbers in AR patients with higher IL-17 and lower TGF-ß1 levels during pollen season [31], while yδT cells correlated negatively with T_reg_ cell percentages and TGF-ß1 levels. **T**_**regs**_ are immune-modulatory cells secreting TGF-ß and IL-10; they suppress Th2 activation by inhibiting IL-4, IL-5, IL-9 and IL-13 production, block effector T cell migration to inflammation sites and further induce IgG4 instead of IgE production by B cells. In nasal fluid of AR patients, lower T_reg_ cell numbers and slightly lower T_reg_ derived TNF-ß levels were found compared to controls [12]. T_reg_ cell-derived IL-10 levels were higher before compared to baseline values after AIT in the same study. Circulating T_reg_ numbers did not differ between healthy subjects and in AR outside of allergen exposure [32], with an increase of T_reg_ cells in nasal mucosa after allergen challenge and during allergen season [33, 34]. Another study described higher circulating T_regs_ numbers in AR patients compared to healthy controls; in the same study T_reg_ numbers remained unchanged after 1 year of AIT compared to baseline values. The FoxP3- IL-10 producing **Tr1 cells**, usually associated with tumor immunity and immune tolerance after organ transplant [35], were reduced in circulating blood and inversely correlated with symptom scores in AR compared to healthy individuals [36]. Naïve CD8+ **T cytotoxic cells** (Tc) have the capacity to produce cytokines involved in allergic inflammation (e.g. IL-4 and IL-5) and stimulate IgE class switching in B cells [37, 38]; successful AIT reduces the IL-4 producing Tc subtype [39]. Recently, an involvement of Tcs in nasal mucosa inflammation of atypical AR patients was also proposed. [40] However, an active role for circulating Tcs in AR has not yet been established. Several studies report reduced blood Tc numbers [41, 42] in allergic airway disease potentially associated with a hyper-releasability of Tc granules [43]. A recent study reported no changes in circulating CD4/CD8 cell ratio in AR patients before or during allergen season compared to controls [22].

In 1995 Pawankar et al. described that T cells in nasal mucosa of AR patients were distinct and did not reflect circulating subsets [44]. The authors concluded that inflammatory cells are compartmentalized in nasal mucosa and circulating blood [45]. Comparison of reported findings regarding circulating lymphocytes is difficult based on differences in study design and heterogeneity of allergen exposure settings. While allergen contact does seem to impact circulating lymphocytes subtypes, particularly on a Th cell subset level, changes in differential blood lymphocyte count are not always observed.

### Innate lymphoid cells

Innate Lymphoid Cells (ILCs), also referred to as natural helper cells, innate helper type 2 cells and nuocytes, are immune cells of the lymphoid lineage, which do not express T or B cell surface markers. Group 2 ILCs (ILC2s) secrete IL-5, IL-9 and IL-13 linking adaptive and innate immune responses in allergic disease. Allergen-associated release of IL-25, IL-33, and TSLP by airway epithelial cells induces ILC2 cytokine secretion and promotion of a Th2 inflammatory response and memory T cell reactivation [46]. In the presence of IL-2, IL-7 or TLSP, ILC2s were also shown to release IL-5 and IL-13 upon stimulation with IL-33 or IL-25 without antigen contact. Their expression of MHC class II molecules and costimulatory signals suggests a role in Th cell activation by antigen presentation [25]. Upon CysLTs and PGD2 stimulation ILC2-derived Th2 cytokines promote airway eosinophilia and mucus hypersecretion [47].

Higher ILC2 numbers in nasal tissue correlated with severity of AR in one study [48]. Dhariwal et al. showed an ILC2 accumulation in the nasal mucosa of AR after allergen challenge; these findings were particularly striking in patients with elevated IgE levels and airway eosinophilia outside of allergy season [49].

Increased ILC2 percentages in circulating blood have been described 4 h after allergen challenge in cat-sensitized AR patients. Allergen challenge in AR rapidly induces increased peripheral blood ILC2s that express CD84 [50]. Similarly, another study described higher circulating ILC2 levels in AR during pollen season, compared to AR subjects after AIT and non-atopic individuals [47, 51]. Interestingly, circulating ILC2s were also higher in HDM sensitized, but not in mugwort sensitized AR patients during allergen season and compared to healthy controls [52]. Circulating ILC2 percentages were similar in AR and in healthy subjects outside of allergen season [53].

Circulating ILC2s are involved in the AR immune reaction and appear to increase in circulation upon allergen exposure, possibly dependent on allergen type.

### B cells

B lymphocytes are activated directly by antigen presenting interaction with T cells, promoting Th2 development by inducing IL-2 cytokine production [54], or indirectly by antigen contact. In AR, nasal mucosa residing IgE-positive B cells can produce IgE [55] and undergo class switch to IgE production upon allergen challenge [56]. IgE also binds on low affinity receptors FcεRII expressed on B cells, further upregulating IgE synthesis.

In blood of AR pollen patients a proportion of allergen-specific B cells showed adaptive memory B-cell responses making them potential sources of allergen-specific IgE upon subsequent allergen exposure [57]. However, the difficult quantification of circulating IgE+ B cells in allergic disease implies a B cell isotype switching to IgE+ B cells in the nasal mucosa through interactions with dendritic cells (DCs) and T cells [58]. A decreased in IgE+ B cells proportion was found in AR patients after AIT without correlation between blood IgE+ B cells and circulating IgE levels [59]. There is evidence that IL-21 regulates IgE levels by binding to B cells in the AR associated immune response [60]. Regulatory B cells (B_regs_) modulate inflammation by IL-10, IL-35 and TGF-ß secretion and are associated with allergen tolerance [61]. Percentages of B_reg_ cells were decreased in AR [26, 61], especially in cases with comorbid allergic asthma.

In summary, in AR circulating B cells appear to be recruited into the nasal mucosa, where class switching to IgE is induced, however, without significantly impact on overall blood B cell numbers.

### Natural killer cells (NK)

NK cells play an important role in mediation between innate and acquired immunity, such as interaction with DCs leading to a selection of antigen-presenting cells. Since the description of subtypes by Wei et al. [62], NK cells are classified into IFN-γ secreting NK1 and IL-4, IL-5 and IL-13 secreting NK2 cells. In AR, higher circulating NK cell percentages and cytotoxicity was shown for IL-4+ NK2 cells but not for IFN-γ + NK1 cells compared with non-atopic controls, correlating with elevated IgE levels [5].

In a murine AR model, depletion of NK cells induced more mucosal and peripheral blood eosinophilia and higher IL-5 levels [63]. In AR patients, NK cells were shown to secrete IL-8 after treatment with IL-15 further indicating a link between NK cells and eosinophils in humans [64]. Circulating NK cells expressing the chemotactant CX3CR1 were increased after nasal allergen challenge in AR, but their relative percentage among lymphocytes remained the same [65].

Reports on circulating NK numbers in AR are scarce; however, the NK2 type appears to be involved in the allergic inflammatory response.

### Monocytes

Depending on their surface receptor expression, circulating monocytes are classified as classical (CD14++CD16−), intermediate (CD14++CD16+) and non-classical monocytes (CD14 + CD16++) [66]. In particular, classical monocytes are shown to differentiate into macrophages or DCs upon homing into tissues.

Most airway macrophages are derived by proliferation of local embryonic precursors; they have homeostatic functions and their numbers remain unchanged after allergen challenge. Blood-derived monocytes, however, can promote allergic inflammation as interstitial macrophages. Macrophages can be subjected to classical activation (M1 cells), induced by IFN-α or lipopolysaccharide (LPS), or alternative activation (M2 cells), induced by IL-4 and IL-13, but also by IL-10, depending on the required pro- or anti-inflammatory cell functions. M2a cells produce pro-allergenic cytokines IL-4 and IL-13 and are involved in different stages of allergic airway disease [67]. In a murine model of AR, lymph node macrophages were associated with IL-4 and IgE production, as well as IgG to IgE immunoglobulin class switching [68]. In human allergic inflammation, gene expression of Th2-related chemokines from recruited CD14+ monocytes in the nasal mucosa was observed simultaneously with accumulation of both mucosal Th2 cells and eosinophils [69]. The authors suggested that recruited monocytes are regulated by IL-4/IL-13 signaling and are directly involved in Th2 pro-inflammatory chemokine production attracting effector cells. Circulating monocytes from atopic patients have a higher FcεRI surface expression correlating with serum IgE levels [70]. Further, higher levels of circulating monocytes have been described in AR compared to healthy controls [71]. Moniuszko et al. reported that circulating monocytes phenotypes differ in HDM AR patients and healthy individuals [72]; CD14++CD16+ and CD14 + CD16++ monocytes subtypes were pronounced in AR patients compared to healthy controls and associated with decreased CD4 + CD25high T cells frequencies. In AR, CD141 monocytes were recruited to the nasal mucosa within hours and DCs accumulated after several days of continued allergen challenge. During allergen season, blood CD14 + monocytes in AR subjects showed higher integrin adhesion molecule (CD11c) surface density compared to asymptomatic but also non-atopic subjects. Glucocorticoids were shown to deplete these subsets potentially limiting allergic inflammation by decreasing antigen presentation [11]. Immune modulating effects have also been described for monocytes. In mice, FcεRI-mediated IgE endocytosis by monocytes induces IgE clearance from serum [73]. After AIT, an increased IL-10 production by monocytes was reported in human AR subjects [74]. Further, a downregulation of monocytes-derived IL-10 by Th2 cytokines has been described in nasal mucosa of AR patients [75].

Circulating monocytes are a source for macrophages and DCs recruited to the nasal mucosa in AR associated inflammation; however, reports on elevated circulating monocyte numbers in AR appear inconsistent.

### Dendritic cells

Antigen-presenting dendritic cells (DCs) in in allergic inflammation are on the one hand involved in pro-allergenic activation of effector T cells, and on the other hand in tolerance induction via activation of T_regs_ cells, such as during allergen immunotherapy. In circulating blood, the two main DC subtypes are CD11c − CD123+ plasmacytoid (pDCs) and CD11c + CD123− myeloid (mDCs) cells; blood DCs are typically without dendrites and less mature compared to tissue DCs. Allergen challenge leads to recruitment of circulating mDCs through chemotactic factors, such as IL-8, RANTES, macrophage inflammatory protein (MIP)-3α and epithelial β-defensins, leading to an increase of mucosal DCs within hours after allergen exposure. In allergic patients, epithelial cells can promote DCs maturation and induction of Th2 polarization by release of TSLP, IL-25 and IL-33 [76].

In allergic asthma, mDCs have increased surface FcɛRI expression levels compared to non-asthmatics, mediate pulmonary inflammation through T-cell and eosinophil activation, and nourish local IgE and Th2 cytokine production [77]. Further, a significant increase of tissue pDC are found in AR after allergen exposure skewing naïve T-cells towards a Th2 phenotype [78] or potentially inducing antigen-specific T-cell subset depletion by selective inhibition [79]. In AR, increased mDCs and pDCs numbers have been demonstrated in nasal mucosa after allergen challenge; further, tissue and blood DCs were shown to express less IL-10, IL-12 and IFN-α in these patients [80]. The authors concluded that these impaired DCs responses in AR patients promote Th2/Th17-cell polarization *in vivo*, since the IL-6/IL-17 pathway was also upregulated by blood mDCs. Another study showed impaired expression of costimulatory molecules by circulating mDCs promoting IL-5 and IL-13 derived Th2 cell responses [81].

AIT promotes DC-mediated naïve T-cells skewing towards an IL-10-producing T_reg_ phenotype and subsequent Th1 response [82]. Indeed, AIT has been shown to result in an increase in IFN-α production from pDCs and elevated circulating IgG4 antibody levels after stimulation with a TLR9 agonist in AR patients [83]. Additionally, local corticosteroids inhibit the production of DC chemotactants in the nasal mucosa disrupting allergen presentation of tissue DCs to effector T cells [79].

While a significant mDCs and slight pDCs decrease was reported in circulating blood 24 h after allergen challenge in allergic asthma patients [84], suggesting their recruitment into the airway mucosa [85], little is known about circulating DCs dynamics after allergen challenge in AR.

### Neutrophils

Migration of neutrophils is dependent on blood IL-8 concentration in AR [86]. While baseline circulating IL-8 levels in AR patients are similar to healthy controls, they were shown to significantly increase after allergen challenge [86]. Further, an increase in circulating IL-6 after allergen challenge has been associated with neutrophil trafficking in atopic AR subjects [49]. IL-17 from Th17 cells was also associated with neutrophil recruitment in AR, probably by inducing IL-8 and CXCL1Groα release by airway fibroblasts. Neutrophils express multiple mediators which mediate airway inflammation such as MMP-9, neutrophil elastase, α-defensin, TGF-ß1 and ROS, supporting eosinophil migration and priming of T cells [87]. The prolonged release of neutrophil elastase and free radicals damage the epithelium and are most likely responsible for vasomotor symptoms characterizing AR. Further, function alterations in ROS generation by circulating blood neutrophils potentially induce (anti)oxidant imbalance resulting in tissue damage [86].

A recent study of nasal cytology in 468 AR patients described a neutrophils dominated (≥90%) inflammatory cell response in 14.32% and a mixed eosinophil/neutrophil (10% ≤ eosinophil < 50%) response in 23.93% of all cases. [88]. Compared to healthy controls higher neutrophil numbers are generally observed in nasal fluid of AR patients [49]. Upon allergen exposure in intermittent AR, particularly early-phase responses are dominated by neutrophil accumulation in nasal mucosa, with positively correlating neutrophil numbers and nasal symptom scores in nasal lavage fluid 1 hour after allergen challenge [89]. Challenge with IL-8 lead to a fast influx of neutrophils into the nasal mucosa measured 30mins and 3 h after challenge [90]. In HDM allergic AR patients late-phase mucosal inflammation also induced circulating neutrophils activation and neutrophils increased in nasal fluid 6 h after allergen challenge [89]. Neutrophils accumulation in nasal mucosa seems partially LT dependent, since montelukast was shown to significantly lower neutrophil numbers 30 min and 6 h after allergen challenge [91].

Blood neutrophils, particularly segmented neutrophils, increase upon allergen challenge [8, 9] and show altered functions in AR. For example, circulating blood neutrophils from AR patients produced more LTB2 after stimulation with calcium ionophore than controls. Also, phagocytic activity of neutrophils was lower in AR subjects compared to healthy controls, but was increased at 7 h and 24 h after allergen compared to baseline. Further, increased ROS generation by circulating neutrophils was found in 24 h after allergen challenge in AR patients [89]. However in the late phase (7 h and 24 h) after allergen challenge neutrophil numbers were not elevated in AR [86].

In summary, circulating neutrophils increase in the early phase after nasal allergen challenge but return to baseline in the late phase. These findings possibly reflect a rapid recruitment of these innate immune cells to the nasal mucosa, where they support effector cell recruitment, but also induce tissue damage in AR.

### Eosinophils

Eosinophils are innate immune system inflammatory cells with a well-known role in allergic inflammation. Their maturation, activation and survival are mainly mediated by IL-5 and eosinophil trafficking by Th2 cytokines, such as IL-5 and IL-13. In allergic inflammation, eosinophils promote Th2 polarization by IL-4, IL-25 and IDO release, but also B cell proliferation and antibody induction. Eosinophils also promote recruitment of Th2 cells by chemotactants such as CCL17/CCL22 and interactions with DCs. Tissue ILC2s have been shown to regulate blood eosinophils by IL-5 secretion [48, 92].

Atopic AR subjects show significantly increased eosinophil numbers in the nasal mucosa compared to healthy subjects [93]. Increased nasal mucosa eosinophils were found days after but not in the first 24 h post allergen challenge in pollen sensitized AR subjects, whereas placebo airway challenge did not induce nasal eosinophilia in AR [33]. Eotaxin levels increased after nasal allergen challenge in AR subjects and were associated with eosinophil and macrophage elevation in nasal fluid and sputum [94]. Eotaxin challenge alone was also shown to induce eosinophilia in nasal fluid of AR [95], without changes in lymphocyte, basophil, and macrophage numbers [96].

Elevated circulating eosinophils have been reported in AR patients during allergen season [97] with positive correlation to clinical symptoms and nasal inflammation. A study in HDM sensitized AR patients revealed a significant increase of eosinophil percentages 24 h after allergen challenge compared to baseline, which was not as pronounced as in allergic asthma [86]. However, this increase in circulating blood eosinophils and their progenitors was not consistently significant, despite regular elevation of plasma IL-5 [98, 99]. Interestingly, in symptomatic AR patients granules of circulating eosinophils were found mostly intact, implying that circulating eosinophils retain their granule contents until they home in target tissues [100]. Also, blood eosinophil chemotaxis and spontaneous ROS production did not significantly differ between AR patients and healthy controls [101].

The role of eosinophils in atopic disease is well known; numbers of circulating blood eosinophils increase in the inflammatory late phase response after allergen exposure in AR, but to a much lesser extent compared to allergic asthma.

### Basophils

Basophils are equipped with surface IgE receptors and release histamine, lipid mediators and cytokines from intracellular vesicles upon IgE cross-linking. In the early phase of the allergic inflammatory response two pathways enable Th2 skewing by basophils: IgE-dependent IL-3, IL-4 and IL-13 secretion after allergen contact and direct stimulation by epithelial cell-derived IL-3, TSLP and IL-33. The late phase of IgE mediated AR responses is mainly induced by basophil-derived LTC4 and histamine following IL-3, IL-5 and GM-CSF activation [102]. IL-17RB expression on ex vivo allergen-challenged basophils from AR patients is IL-3 dependent and was shown to inhibit apoptosis, promoting IgE-mediated basophil degranulation [103]. Allergen-specific reactivity of circulating basophils was found to exhibit a Clock dependent circadian variability in seasonal AR [104].

In AR patients, basophil accumulation in nasal mucosa was described [105, 106], associated with subsequent histamine release 11 h after allergen challenge [105]. An increase in IL-18 levels was shown 4 h after nasal allergen challenge and correlated with basophil proportions after 24 h. [57] Another study found increased nasal mucosa basophils 1 hour after allergen challenge which persisted up to 1 week [107]. IL-33 protein was expressed by AR nasal epithelial cells in response to ragweed pollen exposure; IL-33 supported mucosal accumulation of eosinophils and basophils along with chemoattractant production from FcεRI1 expressing mast cells and basophils [108]. Further, nasal challenge with RANTES induced an allergic mucosal response with eosinophils and basophils infiltration [109].

Nasal allergen challenge was associated with increased blood basophil activation marked by peaks in circulating basophil activation markers up until 24 h after challenge [110] and spontaneous secretion of IL-13 [4]. Blood basophil sensitivity correlated with allergen-specific IgE fraction and was associated with clinical symptoms during grass pollen season [111]. In an early pioneer study, Pedersen et al. found that rhG-CSF treatment increases circulating basophils numbers, but reduces average basophil histamine content and releasability [112]. Persistent suppression of basophil response was associated with lower clinical symptoms years after successful grass pollen AIT [112, 113]. Reduced basophil responsiveness after AIT was confirmed by another study, implying that IgG antibodies compete with allergen for blocking of basophil surface FcεRIIb receptors [114]; However, whole blood basophils did not prove to be sufficient biomarker for adverse effects or early clinical responses onset in AIT [115].

Despite reports of circulating basophil decrease in allergic asthma [104, 116], possibly due to recruitment to the airway mucosa in the late phase response, changes in blood basophil numbers are not consistent in AR. However, basophil responsiveness seems to be associated with allergic inflammatory responses in AR and therapeutic success of AIT.

### Thrombocytes

Beside their established role in coagulation, findings from animal studies with induced platelet depletion have shown that thrombocytes are also clearly involved in allergic disease [117, 118]. In human subjects, low-affinity and high-affinity IgE receptors on the membrane of platelets, as well as TLR2, TLR4, TLR9 innate immune receptors provide a potential link to allergic inflammatory responses. [119]. Platelets form complexes with leukocytes, supporting their recruitment into tissues in response to inflammatory stimuli; circulating platelet-leukocyte complexes, displaying α-M subunits (CD11b), which promote leukocyte attachment to the vascular epithelium, have been described [117]. A similar finding has been reported for platelet P-selectin associated promoting of endothelial eosinophil attachment [120]. CD40 ligand on activated platelets promotes antigen presentation to the adaptive immune response inducing DCs maturation and Th2 polarization responses following allergen challenge [121]. In allergic asthma, platelets have been shown to undergo chemotaxis after allergen exposure in vitro [122] and also contribute to acute bronchoconstriction, bronchial hyper-reactivity, immune cell infiltration and airway remodeling [6]. In lethal *status asthmaticus* increased megakaryocytes numbers were found in lung circulation [123].

Data on circulating thrombocyte dynamics during allergen challenge in AR are lacking, but an impairment of platelet aggregation correlating with IgE levels was previously shown [124]. Serum PF-4 and β-TG levels in HDM AR patients appear to be comparable to those in healthy controls [125]. Incidentally, we found a significant increase in circulating thrombocytes after 2 h of continuous allergen challenge compared to baseline values [8, 9]; however, 4 h after allergen challenge, no significant changes in circulating thrombocyte numbers were observed (data not published). During AIT in grass pollen AR, no changes in platelet activation marker β-TG levels were observed in plasma, even with during administration of the highest vaccine dose [126].

Little is known about circulating platelets in AR. Analogous to findings in allergic asthma, recruitment of circulating platelets to airway mucosa in the early phase of AR with subsequent support of effector cell adhesion and extravasation into the inflammation site is possible, but remains to be evaluated.

### Erythrocytes

While the main role of red blood cells (RBC) is oxygen transportation, their crosstalk with immune cells has recently gained interest. DAMPs such as heme, Hsp70 and IL-33 have been identified in RBCs [127, 128], which are released into circulation upon intravascular hemolysis. If not neutralized by scavenger proteins, RBC-derived DAMPs can potentiate systemic inflammatory responses. In a model of allergy-induced anaphylaxis [129] a decrease in circulating RBCs was observed as a potential result of aggregation of erythrocytes, leucocytes and platelets; RBC adhesion to activated neutrophils and platelets might cause thrombosis in lowered blood flow settings and hypoxia [129, 130]. Anaphylaxis-associated hypoxia has been shown to result in a H2O2 release from RBCs leading to neutrophils chemotaxis [131].

An involvement of erythrocytes in the allergic immune response has not yet been established. In AR subjects, free hemoglobin has been found in nasal lavage after allergen challenge (micro-epistaxis), possibly as a result of increased vascular permeability [132]. We recently reported significant decreases of circulating RBCs and hematocrit in AR after 2 h, 4 h and 6 h of continuous allergen exposure in a specialized challenge chamber [8, 9]. Due to the concomitant increase in segmented neutrophils, we hypothesized a mechanical trapping of circulating erythrocytes in the airway capillaries by NETs. LT-induced eryptosis during the acute allergic inflammatory response could potentially contribute to this highly significant circulating RBC decrease after allergen challenge.

Taken together, decrease of erythrocytes during the early allergic immune response in AR has been observed. A contribution of RBCs to inflammation by release of DAMPs and ROS for neutrophil chemotaxis remains to be evaluated in mechanistic studies.

## The cellular orchestra in AR

**Fig. 1:**
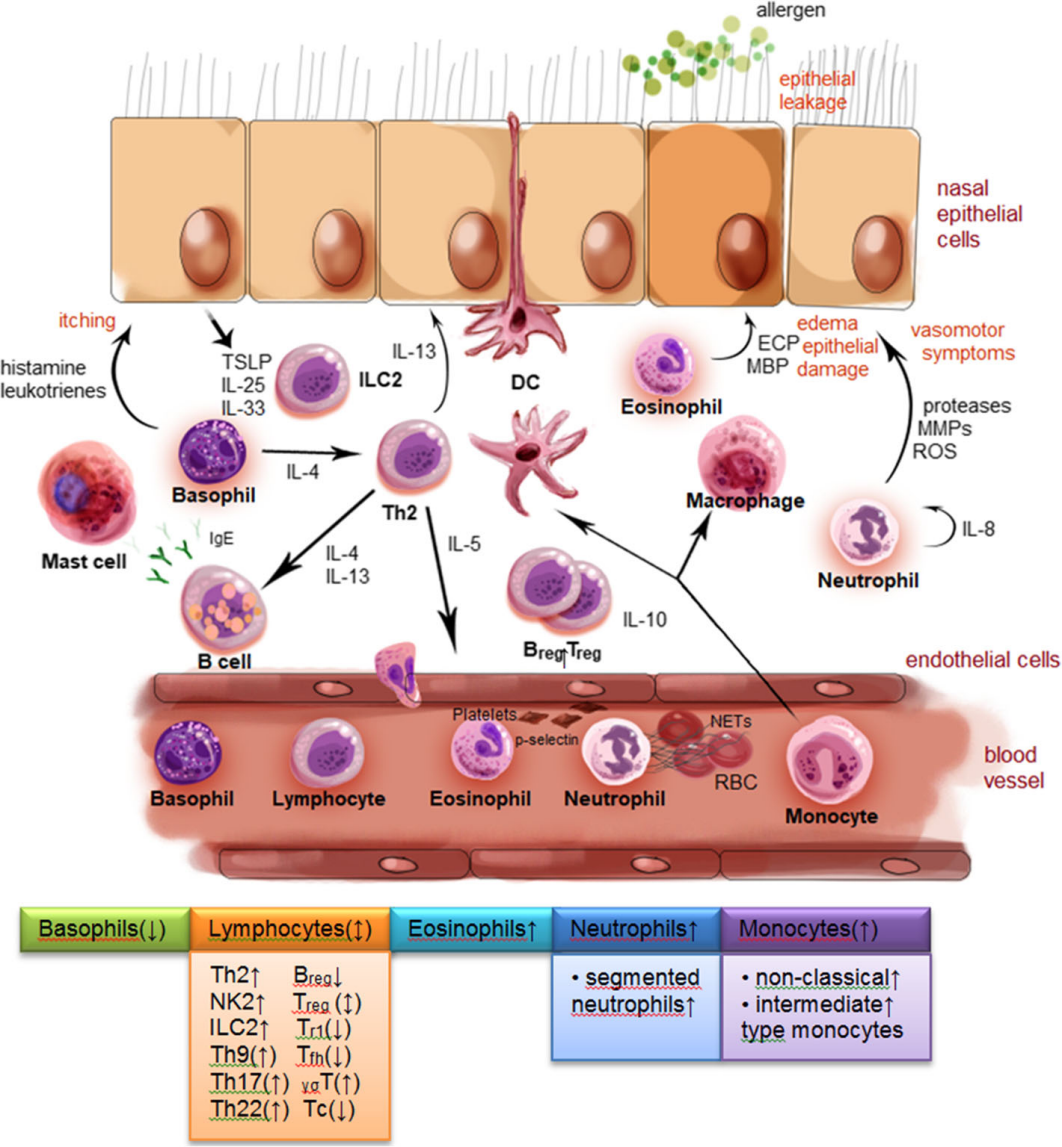
How circulating blood cells participate in allergic rhinitis (AR). Allergen exposure initiates a local inflammatory response involving recruitment of circulating blood cells to the nasal mucosa. In the early phase, neutrophils are mobilized as first-line responders of the innate immune system and contribute to epithelial and nerve damage via cytotoxic mediator release. Damaged epithelial cells release DAMPs and cytokines (e.g. TSLP, IL-25, IL-33) recruiting circulating lymphocyte subtypes (e.g. ILC2), which accumulate in the nasal mucosa and promote the Th2 inflammatory response. In the late phase, mediator release from recruited eosinophils and basophils further contributes to AR symptoms via epithelial damage and microvascular leaking. Blood-derived monocytes participate in promotion and resolution of the allergic response by differentiation into DCs and tissue macrophages. Boxes below the cartoon illustrate different blood cells and their reported changes after allergen exposure. DC, dendritic cell; ECP, eosinophil cationic protein; ILC, Innate Lymphoid Cell; MBP, major basic protein; MMP, matrix metalloprotease; NET, neutrophil extracellular trap; RBC, red blood cell; ROS, reactive oxygen species; for simplicity, IgE-mediated mechanisms are not illustrated

Upon allergen encounter there is a pull of circulating blood cells to the local allergic reaction site in the nasal mucosa in AR (Fig. 1). Neutrophils are recruited to the nasal mucosa in the early phase of the inflammatory response as first-line defense of the innate immune system; beside direct damage induced by certain allergens (e.g. with enzymatic properties), neutrophil-derived cytokines and release of cytotoxic mediators support epithelia damage and nerve ending disturbance (edema, rhinorrhea, vasomotor symptoms). Specific circulating lymphocyte subtypes (e.g. ILC2) accumulate in the nasal mucosa based on cytokines released by damaged epithelial cells (e.g. TSLP, IL-25, IL-33) and Th2 cytokines, which further lead to eosinophil maturation, recruitment and survival in the late phase contributing to further epithelial damage and microvascular leaking. Basophils influx amplifies IgE-mediated mediator release (e.g. histamine, leukotrienes) supporting symptomatic inflammation along with local mast cells. Blood monocytes functionally differentiate into DCs and tissue macrophages, thus participating in the promotion but also in the resolution of the Th2 inflammatory response. After allergen immunotherapy, B_regs_ and T_regs_ access the nasal mucosa and initiate immune-modulation via IL-10 release and induction of antibody class switching from IgE to IgG.

## Conclusions

For all of the reasons discussed above, the view on the nasal mucosa as an isolated organ has to be corrected. Multiple evidence for the involvement of circulating blood cells is provided for all phases of the allergic inflammatory response (Table 1). Overall, *in vivo* reports are consistently reporting an early increase in circulating segmented neutrophils followed by a delayed elevation in peripheral ILC2 and eosinophils numbers upon allergen exposure in AR, implying their recruitment in the nasal mucosa. Besides the resulting elevation in circulating leukocytes, a rapid decrease in erythrocytes and, to a lesser extent, changes in thrombocyte numbers reflect a systemic impact of the acute inflammatory AR reaction. Regarding the effects of AIT, the most striking associations between blood cell numbers and therapy success seem to be changes in circulating T cell subsets and their respective cytokines, namely lower Th2, Th9 and Th17 in favor of higher Th1 and T_reg_ cell numbers.

**Table 1:** Circulating blood cells involved in AR-associated inflammation

Cell Type	Subset	Main mediators	Established role	Findings in allergic rhinitis (AR)
T cells	Th2	• IL-4 • IL-5 • IL-13 • IL-10	• extracellular parasites • allergic inflammation	• nasal Th2 cytokine predominance • ↑ circulating Th2 cells in AR • ↓ circulating numbers after AIT
	Th9	• IL-9 • IL-10	• extracellular pathogens • allergic inflammation	• Th9 cytokines associated with nasal eosinophil infiltration and survival in mice • (↑) circulating Th9 cells in AR • (↓) circulating Th9 cells after AIT
	Th17	• IL-17A • IL-17F • IL-21 • IL-22 • CCL20	• extracellular bacteria • fungi • autoimmune disease	• Th17 cytokines associated with nasal eosinophil and neutrophil infiltration • ↑ serum IL-17A levels • (↑) circulating Th17 cells in AR • (↓) circulating Th17 numbers after AIT
	Th22	• IL-22	• proinflammatory and immune-modulating functions • wound healing • cell proliferation • anti-apoptosis	• correlation with clinical symptoms • ↑ circulating Th22 numbers in HDM AR
	T_fh_	• IL-21	Promotion of • germinal center responses • B cell class switching	• IL-4 source (?) • inducer of Th2 cell responses • (↓) circulating T_fh_ cells in AR
	Cytotoxic T cell (Tc)	• perforin • protease • IFN-γ	• intracellular pathogens • induced cell apoptosis	• involved in atypical AR (?) • stimulate B cell IgE class switching • (↓) circulating Tc cells in AR • ↓ IL-4 producing subtype after AIT
	γδ T cell	• IFNγ • IL-17A • IL-17F • IL-22	• proinflammatory and immune-modulating functions at epithelial surfaces • innate and adaptive immunity participation	• yδT cytokines induce B cell IgE synthesis • support Th2 inflammatory response • oligoclonal proliferation in nasal mucosa (?) • ↑ circulating yδT cell percentages correlating with Th17 numbers in AR • negative correlation with T_regs_
	Tr1	• IL-10	• immune-modulating	• inverse correlation with symptom scores • (↓) circulating Tr1 cells in AR
	T_reg_	• IL-10 • TGF-β • IL-35	• immune tolerance • immune modulatory • lymphocyte homeostasis	• ↑ T_regs_ in nasal mucosa after allergen exposure • ↨ circulating T_reg_ numbers in AR • ↔ circulating T_regs_ 1 year after AIT
B cells	B cell		• antibody production • antigen presentation • IgE source in allergic disease	• circulating allergen-specific B cells show adaptive memory responses • isotype switching to IgE+ B cells in nasal mucosa through interactions with local dendritic and T cells (?) • (↓) circulating IgE+ B cells after AIT without IgE correlation
	B_reg_	• IL-10	• antibody production (↑IgG4) • immune modulation • IgG4 source in AIT	• ↓ circulating B_regs_ in AR especially in comorbid allergic asthma
Natural Killer Cells (NKs)	NK	• perforin • proteases • α-defensin	• cytotoxic • intracellular viruses • tumor cell clearance	• NK2 involved in effector cell chemotaxis (?) • ↑ circulating NK2 cells correlating with IgE levels in AR
	NK2	• IL-4 • IL-5 • IL-13		
Innate Lymphoid Cells (ILCs)	ILC2	• IL-4 • IL-5 • IL-9 • IL-13	• allergic inflammation • atopic conditions	• nasal ILCs numbers associated with disease severity in AR • ↑ nasal ILC2 after allergen challenge in AR • (↑) circulating ILC2 percentages after allergen challenge in AR • ↔ circulating ILC2 outside allergen season in AR
Monocytes		• IL-1β • IL-6 • IL-10 • TNF-α	• pathogen defense • phagocytosis • antigen presentation • differentiation into macrophages or dendritic cells	• classical monocytes (CD14++CD16−) source for interstitial macrophages (?) • attracting effector cells • (↑) integrin adhesion molecule (CD11c) surface density in AR • monocyte-derived IL-10 downregulation by Th2 cytokines • (↑) circulating non-classical CD14++CD16+ and intermediate CD14 + CD16++ monocyte levels in AR • ↓ antigen presentation capacity after glucocorticoids • (↑) increased IL-10 production after AIT
Dendritic Cells	pDC mDC		• antigen presentation • activation of effector T cells • tolerance induction via activation of T_regs_	• mucosal pDC and mDCs ↑ after allergen exposure in AR • mDCs have ↑ surface FcɛRI levels in AR • blood DCs express ↓ IL-10, IL-12 and IFN-α in AR (Th2 promotion?) • AIT ↑ DCs mediated naïve T-cells skewing towards IL-10-producing T_regs_ • AIT ↑ IFN-α production from pDCs • local corticosteroids disrupt allergen presentation of mucosal DCs
Eosinophils		• MBP • ECP • EPX • EDN	• helminth defense • allergic inflammation	• ↑ in nasal mucosa after allergen challenge • circulating eosinophils degranulate in target tissues • correlation with clinical symptoms eotaxin associated (?) • ↑ circulating eosinophil numbers in the late phase after allergen exposure in AR
Basophils		• histamine • serotonin • tryptase • PGD2 • LTC4 • PAF	• parasite defense • allergic inflammation	• enable IgE-dependent Th2 skewing after allergen contact • FcεRI1 expression • ↑ accumulation in nasal mucosa associated with clinical symptoms • (↓) circulating basophil numbers, but (↑) activation markers after allergen challenge in AR • (↓) responsiveness after AIT (IgG antibodies mediated basophil FcεRIIb blocking)
Neutrophils	Segmented neutrophil	• MMP-9 • elastase • α-defensin • TGF-ß1 • ROS	• first-line innate immune responses against pathogens • release of neutrophil extracellular traps (NETs) • phagocytosis	• supporting eosinophil migration and T cells priming • mediators associated with vasomotor symptoms • altered functions in LTB2 production, ROS generation and phagocytic activity in AR • ↑ circulating neutrophils in early phase after allergen challenge • ↓ nasal accumulation after montelukast therapy
Thrombocytes (Platelets)			• coagulation	• impaired aggregation correlating with IgE (?) • P-selectin mediated vascular attachment of leukocytes (?) • induction of DCs maturation and Th2 polarization (?)
Erythrocytes (RBCs)			• respiratory gas exchange	• ↓ circulating RBCs in early phase after allergen challenge in AR • neutrophil chemotaxis by DAMPs (heme, Hsp70 and IL-33) and ROS release (?)

↓ higher; ↑ lower; ↔ unchanged; (↑) inconsistent reports of higher numbers, (↓) inconsistent reports of lower numbers, (↨) reports of higher and lower numbers; AIT allergen immunotherapy, mDCs myeloid Dendritic Cells, pDCs plasmacytoid Dendritic Cells, ECP eosinophil cationic protein, EDN eosinophil-derived neurotoxin, EPX eosinophil peroxidase, LT leukotriene, NK Natural Killer Cell, ILC Innate Lymphoid Cell, MBP major basic protein, MMP matrix metalloprotease, RBC red blood cell, ROS reactive oxygen species; (?) suspected, or controversial studies ongoing

## Abbreviations

AIT: Allergen-specific immunotherapy
AR: Allergic rhinitis
BCR: B cell receptor
B_reg_: Regulatory B cell
CSF: Colony stimulating factor
CysLT: Cysteinyl leukotriene
DAMPs: Damage-associated molecular patterns
DCs: Dendritic cells
HDM: House dust mite
IFN: Interferon
IL: Interleukin
ILC: Innate lymphoid cell
LT: Leukotriene
NET: Neutrophil extracellular trap
NK: Natural killer cell
RBC: Red blood cell
ROS: Reactive oxygen species
Tc: T cytotoxic cell
TCR: T cell receptor
Tfh: T follicular helper cell
TGF: Transforming growth factor
Th: T helper cell
Th0: Naïve T helper cell
T_reg_: Regulatory T cell
TSLP: Thymic stromal lymphopoietin
